# De novo mtDNA point mutations are common and have a low recurrence risk

**DOI:** 10.1136/jmedgenet-2016-103876

**Published:** 2016-07-22

**Authors:** Suzanne C E H Sallevelt, Christine E M de Die-Smulders, Alexandra T M Hendrickx, Debby M E I Hellebrekers, Irenaeus F M de Coo, Charlotte L Alston, Charlotte Knowles, Robert W Taylor, Robert McFarland, Hubert J M Smeets

**Affiliations:** 1 Department of Clinical Genetics, Maastricht University Medical Centre (MUMC), Maastricht, The Netherlands; 2 Research School for Developmental Biology (GROW), Maastricht University, Maastricht, The Netherlands; 3 Department of Neurology, Erasmus MC-Sophia Children's Hospital Rotterdam, Rotterdam, The Netherlands; 4 Wellcome Trust Centre for Mitochondrial Research, Institute of Neuroscience, The Medical School, Newcastle University, Newcastle upon Tyne, UK; 5 Research School for Cardiovascular Diseases in Maastricht, CARIM, Maastricht University, Maastricht, The Netherlands

**Keywords:** mDNA mutations, de novo, prenatal diagnosis (PND), genetic counselling

## Abstract

**Background:**

Severe, disease-causing germline mitochondrial (mt)DNA mutations are maternally inherited or arise de novo. Strategies to prevent transmission are generally available, but depend on recurrence risks, ranging from high/unpredictable for many familial mtDNA point mutations to very low for sporadic, large-scale single mtDNA deletions. Comprehensive data are lacking for de novo mtDNA point mutations, often leading to misconceptions and incorrect counselling regarding recurrence risk and reproductive options. We aim to study the relevance and recurrence risk of apparently de novo mtDNA point mutations.

**Methods:**

Systematic study of prenatal diagnosis (PND) and recurrence of mtDNA point mutations in families with de novo cases, including new and published data. ‘De novo’ based on the absence of the mutation in multiple (postmitotic) maternal tissues is preferred, but mutations absent in maternal blood only were also included.

**Results:**

In our series of 105 index patients (33 children and 72 adults) with (likely) pathogenic mtDNA point mutations, the de novo frequency was 24.6%, the majority being paediatric. PND was performed in subsequent pregnancies of mothers of four de novo cases. A fifth mother opted for preimplantation genetic diagnosis because of a coexisting Mendelian genetic disorder. The mtDNA mutation was absent in all four prenatal samples and all 11 oocytes/embryos tested. A literature survey revealed 137 de novo cases, but PND was only performed for 9 (including 1 unpublished) mothers. In one, recurrence occurred in two subsequent pregnancies, presumably due to germline mosaicism.

**Conclusions:**

De novo mtDNA point mutations are a common cause of mtDNA disease. Recurrence risk is low. This is relevant for genetic counselling, particularly for reproductive options. PND can be offered for reassurance.

## Introduction

Mitochondrial diseases due to defective oxidative phosphorylation are the most common inborn errors of metabolism,^1^ with between 15% and 25% of cases caused by pathogenic mitochondrial (mt)DNA mutations.^1^ ^2^ In the majority of cases, these mtDNA mutations are heteroplasmic, a mixture of mutated and wild-type mtDNA molecules in cells and/or tissues of an individual. At a certain level of mtDNA mutant load, the cell expresses dysfunction and symptoms will occur, the so-called threshold effect. This threshold varies within tissues and between different mutations and is difficult to specify for most mtDNA mutations. Nevertheless, mutant loads of below ∼18% are not considered to cause symptoms in >95% of the cases.^3^ Severity of clinical involvement broadly increases with a higher heteroplasmy level, although clearly this is not the only factor. Individuals exclusively receive their mtDNA from their mother. It is transmitted, at least in part, through a genetic bottleneck induced by a drastic reduction in the number of mtDNA molecules per primordial germ cell during oogenesis, leaving a few mtDNAs to become the founders for the offspring, although the exact nature and timing of events remain topic of debate.^4–8^ The result is, however, indisputable: children of a woman carrying a heteroplasmic mtDNA mutation can display a wide variety of mutation loads.

Disease-causing mtDNA mutations can be point mutations or single, large-scale deletions/rearrangements as a primary cause, or multiple deletions and depletions usually secondary to a nuclear gene defect or environmental factor (eg, nucleoside RT inhibitors and ageing). The recurrence risk in a subsequent pregnancy depends on the underlying primary genetic defect. The potential of a severe phenotype and the lack of effective treatment often prompts couples who have affected offspring or a positive family history of mtDNA disease to request intervention to prevent transmission.

Women harbouring an mtDNA deletion have a low risk (∼1 in 24) of clinically affected offspring,^9^ whereas females with an mtDNA point mutation potentially have a high risk of recurrence. Often, for female carriers the risk of having an affected child is difficult to predict. However, a substantial proportion of the children with a single, large-scale mtDNA deletion or point mutation have a de novo mutation not inherited from their mother.^1^ The recurrence risk of de novo, single large-scale mtDNA deletions in subsequent offspring is low.^9–11^ For de novo point mutations, one would expect the recurrence risk to be similarly low, although this has not been systematically investigated to date.

In reproductive counselling of mtDNA mutations, the choice between prenatal diagnosis (PND) and preimplantation genetic diagnosis (PGD) depends mainly on the recurrence risk and on the expected predictive value of the test. The latter can be problematic in PND given the potential difficulties of interpreting results for most mtDNA mutations when fetal mutant load falls within a ‘grey zone’ where correlation between genotype and phenotype is unclear. However, when the likelihood of offspring with either no mutation or a mutant load below the heteroplasmic threshold of disease expression is high, PND can be applied for reassurance. This is the case for single, large-scale mtDNA deletions^9–12^ and for low-level mtDNA mutations demonstrating skewing to the extremes (eg, m.8993T>G) in the mother.^13^ Also, when maternal mutation load of a non-skewing mtDNA mutation is very low, PND could be considered. For familial mtDNA mutations showing an unpredictable and/or high recurrence risk, PGD is an attractive option,^3^ ^14^ although patient preference and choice plays an important role in the decision-making process. Moreover, other factors including maternal age or pre-existent fertility problems may influence an informed decision regarding preferred reproductive options.

In order to study the relevance and recurrence risk of apparently de novo mtDNA point mutations, we evaluated the occurrence of de novo mtDNA point mutations in our own experience and in the literature, and present data on five couples presenting to our own clinical services who have had an affected child and for whom PND or PGD has been performed in a subsequent pregnancy. We offer recommendations for reproductive counselling including strategies to prevent the birth of children affected by mtDNA disease.

## Materials and methods

### Patients and patient data

The frequency of de novo versus recurrent mtDNA disease was studied by cataloguing all (likely) pathogenic mtDNA point mutations identified in our diagnostic laboratory either by specifically screening for known mutations (m.3243A>G, m.8344A>G, m.8993T>C/G) or sequencing the entire mitochondrial genome. ‘Likely’ pathogenic refers to novel mutations that are suspected to be pathogenic based on well-established prediction tools and accepted criteria;^15^ ^16^ however, this was not in all cases proven by functional (eg, single-fibre segregation, *trans*mitochondrial cybrid) studies. In addition, we documented whether maternal relatives of the index patients were tested to determine presence and, if applicable, heteroplasmy levels of the mtDNA mutation. Apparent de novo mtDNA mutations were defined by their absence in one or more accessible maternal tissues (eg, blood, urinary sediment, buccal epithelia) and, if tested, absence in other matrilineal relatives. Conversely, if the mtDNA mutation was detected in the mother and/or other maternal relatives, it was classified as maternally inherited/familial. The de novo frequency was calculated by taking the proportion of apparently de novo mutations from all mtDNA mutations identified between January 1996 and March 2015. Cases where it was not clear whether the reported mtDNA mutation was (apparently) de novo or maternally inherited/familial because additional familial testing was not possible were excluded from the analysis.

### Literature search

PubMed was systematically searched as of December 2015 for cases of de novo mtDNA mutations. Search terms were ‘mtDNA de novo mutation’. PubMed's automated query translation, incorporating MeSH terms and enhancements from the Unified Medical Language System (UMLS), was as follows: “dna, mitochondrial"[MeSH Terms] OR (“dna"[All Fields] AND “mitochondrial"[All Fields]) OR “mitochondrial dna"[All Fields] OR “mtdna"[All Fields]) AND “de novo"[All Fields] AND (“mutation"[MeSH Terms] OR “mutation"[All Fields]. Also ‘related citations’ of de novo reports in PubMed were screened.

### Prenatal diagnosis and preimplantation genetic diagnosis

Chorionic villus sampling (CVS) samples were obtained at 10+4 weeks and 11+2 weeks gestation, respectively, amniotic fluid sampling at 16+2 weeks and 16+6 weeks, respectively. DNA extraction from prenatal and postnatal tissues, quantitative analyses of the mtDNA mutations as well as the PGD procedure were performed as previously described.^14^ ^16^ ^17^ Primers used for the Maastricht cases are for m.8993T>C/G: CACACCTACACCCCTTATCCC (forward) and TCATTATGTGTTGTCGTGCAG (reverse); for m.5556G>A: CACCATCATAGCCACCATCA (forward) and GGCTGAGTGAAGCATTGGAC (reverse); for m.8969G>A: GCTTCATTCATTGCCCCCAC (forward) and AGGGCTATTGGTTGAATGAGTAAG (reverse); and for m.3243A>G: CAACTTAGTATTATACCCACAC (forward) and TTTCGTTCGGTAAGCATTAG (reverse). Mutation-specific restriction enzymes are *HpaII* (Roche) for m.8993T>C/G, *DdeI* (Roche) for m.5556G>A, *AluI* (Roche) for m.8969G>A and *HaeIII* (Roche) for m.3243A>G. For the Newcastle cases (ref. 18 and unpublished data), pyrosequencing was used to quantitatively assess mtDNA mutation heteroplasmy levels as described previously^19^ (primer sequences available on request). For one patient, further assessment of mtDNA heteroplasmy was undertaken using next-generation sequencing (NGS)-based mtDNA sequencing; briefly, patient DNA samples were amplified by long-range PCR, sheared using the Ion Xpress Plus Fragment Library Kit and sequenced on an Ion Torrent PGM platform according to manufacturer’s protocols (Life Technologies, Foster City, California, USA).

## Results

### De novo mtDNA point mutations

In our diagnostic laboratory, 105 index cases were identified based on laboratory diagnoses with a (likely) pathogenic mtDNA point mutation (table 1 and online supplementary table S1). The majority (72/105) being adults, our cohort seems a fair representation of the patient population. A subset of 17 patients were found to harbour an apparently de novo mtDNA mutation (table 1), of which 12 were between 0 and 3 years of age at investigation, whereas 5 were >18 years old. Of note, in 3 of 17 cases only maternal blood was analysed, and with a semiquantitative method. In two other mothers, besides blood only hair, and hair plus fibroblasts, respectively, were tested. These cases have a lower probability of being truly de novo than the remaining 12 where besides blood also maternal urine and/or muscle has been investigated. Maternal inheritance was firmly established for 52 patients, 35 of whom were adults (see online supplementary table S1). Notably, for one of these cases (patient 44) we were able to demonstrate de novo occurrence of the pathogenic mtDNA mutation in the mother of the patient, which might also be the case for two other families (families 14 and 20). For the outstanding 36 patients, it remains unknown whether the mtDNA mutation arose de novo in the index patient or not (see online supplementary table S1). Accordingly, 17/69 (24.6%) of the (likely) pathogenic mtDNA point mutations occurred de novo in the index patient in our series. Additionally, we identified a further 137 de novo cases in the literature (see online supplementary table S2; those published from our own centre and therefore shown in table 1 or online supplementary table S1 are not included). These are listed according to whether the mother and/or siblings were tested as well, and if so, whether this was carried out in one or more tissues to assess mtDNA heteroplasmy levels. Reports with apparently de novo mtDNA mutations where no (close/relevant) relatives were tested^20–24^ are not included in the table.

**Table 1:** (Likely) Pathogenic mtDNA mutations identified in our diagnostic laboratory (Maastricht), presumably de novo in the index patients

	Reference	Family no.	Gene	Mutation	Mutation load(s) in tested tissue(s) of index patient	Mutation load(s) in tested tissues of (maternal) relative(s)	Index patient's age at investigation
*De novo cases*							
1.	–	16390	*MTTL1*	m.3243A>G	12% (Bl)	Mother: n (Bl, U) Daughter: 4% (U)	44
2.	–	19462	*MTTL1*	m.3243A>G	8% (M, Bl)	Mother: n (Bl, U)	3
3.	This article (case 5)	22023	*MTTL1*	m.3243A>G	13% (Bl), 12% (M), 17% (F), 16% (U), 14% (BM)	Mother: n (Bl, M, BM) 11 oocytes/embryos in PGD cycle:	2
4.	This article (case 2)	15503	*MTTW*	m.5556G>A	>90% (M) (not tested in our laboratory)	n Mother: n (Bl, H, U, M) Mother's subsequent pregnancy: n (amniocentesis)	0
5.	This article (case 3)	17063	*MTATP6*	m.8969G>A	95% (Bl, F, M)	Mother: n (Bl, U) Mother's subsequent pregnancy: n (amniocentesis)	0
6.	This article (case 1)	7387	*MTATP6*	m.8993T>G	90% (M)	Mother: n (Bl, H, M) Mother's subsequent pregnancy: n (CVS)	1
7.	This article (case 4)	19006	*MTATP6*	m.8993T>G	97% (Bl, M), 96% (F)	Mother: n (Bl, U, H) Mother's subsequent pregnancy: n (abortus material) Mother's second subsequent pregnancy: n (CVS)	0
8.	–	21838	*MTATP6*	m.8993T>G	92% (M), 90% (Bl)	Mother: n (Bl, U)	1
9.	–	14652	*MTATP6*	m.9155A>G	88% (M)	Mother: n (Bl, M)	1
10.	–	9868	*MTND3*	m.10191T>C	100% (Bl, M)	Mother: n (Bl, M, H, U)	0
11.	–	2869	*MTTS2*	m.12207G>A	>60% (M), n (Bl) (with semiquantitative sequence analysis)	Mother: n (Bl) (with semiquantitative sequence analysis)	41
12.	Blok *et al*^49^	6604	*MTND5*	m.13511A>T	65% (Bl), 53–65% (F), 72% (M)	Mother: n (M, Bl, H)	3
13.	Blok *et al*^49^	2339	*MTND5*	m.13513G>A	4–6% (Bl), 13–15% (M), 1–5% (F)	Mother: n (Bl, F, H) Two sisters: n (Bl) Maternal grandmother: n (Bl, F)	19
14.	Blok *et al*^49^	4707	*MTND5*	m.13513G>A	11–16% (Bl), 17% (H), 16% (M), n (F)	Mother: n (Bl, H) Maternal grandmother: n (Bl, H)	1
15.	–	18686	*MTND5*	m.13513G>A	1% (Bl), 10% (M)	Mother: n (Bl, M)	42
16.	–	22006	*MTCYB*	m.15153G>A	Heteroplasmic (Bl, M) (with semiquantitative sequence analysis)	Mother: n (Bl) (with semiquantitative sequence analysis)	43
17.	–	27171	*MTCYB*	m.15158A>G	Heteroplasmic (Bl, M) (with semiquantitative sequence analysis)	Mother: n (Bl) (with semiquantitative sequence analysis)	0

Mutations are listed according to nucleotide position.

Bl, blood; BM, buccal mucosa; CVS, chorionic villus sampling; F, fibroblasts; H, hair; M, muscle; n, normal (mutation not detected); PGD, preimplantation genetic diagnosis; U, urine.

10.1136/jmedgenet-2016-103876.supp1 supplementary tables

### PND/PGD in subsequent pregnancies

**Figure 1:**
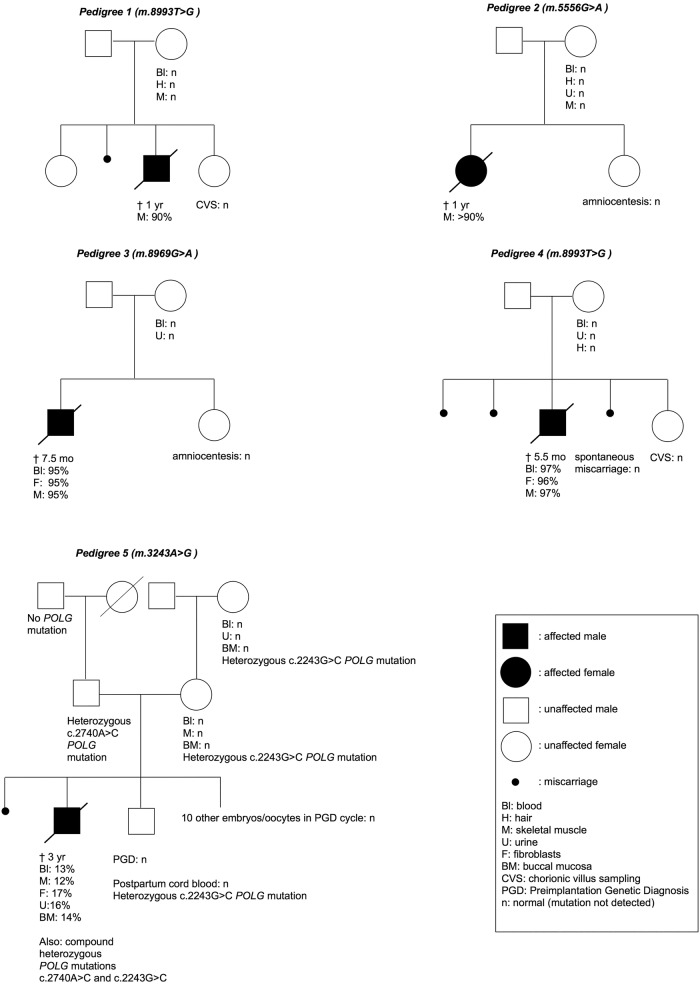
Pedigrees of the five case descriptions in this report.

The parents of four of the five children described below with apparently de novo mtDNA disease (figure 1, tables 1 and 2) were counselled in the outpatient department of Clinical Genetics in Maastricht. For case 3, the parents were counselled at another centre while mtDNA analyses were performed in Maastricht.

**Table 2:** Presumably de novo mtDNA mutations for which prenatal diagnosis (PND) and/or preimplantation genetic diagnosis (PGD) has been performed in a subsequent pregnancy (Maastricht+Newcastle+literature)

	Reference	Gene	Mutation	Mutation load(s) in tested tissue(s) of index patient	Mutation load(s) in tested tissues of (maternal) relative(s)
1.	This article (case 1)	*MTATP6*	m.8993T>G	90% (M)	Mother: n (Bl, H, M) Mother's subsequent pregnancy: n (CVS)
2.	This article (case 2)	*MTTW*	m.5556G>A	>90% (M)	Mother: n (Bl, H, U, M) Mother's subsequent pregnancy: n (amniocentesis)
3.	This article (case 3)	*MTATP6*	m.8969G>A	95% (Bl, F, M)	Mother: n (Bl, U) Mother's subsequent pregnancy: n (amniocentesis)
4.	This article (case 4)	*MTATP6*	m.8993T>G	97% (Bl, M), 96% (F)	Mother: n (Bl, U, H) Mother's subsequent pregnancy: n (abortus material) Mother's second subsequent pregnancy: n (CVS)
5.	This article (case 5)	*MTTL1*	m.3243A>G	13% (Bl), 12% (M), 17% (F), 16% (U), 14% (BM)	Mother: n (Bl, M, BM) 11 oocytes/embryos in PGD cycle: n
6.	Lebon *et al*^37^	*MTND3*	m.10158T>C	85% (M)	Mother: n (Bl) Mother's subsequent pregnancy: n (CVS and amniocentesis)
7.	Steffann *et al*^38^	*MTATP6*	m.8993T>G	90% (Bl)	Mother: n (Bl) Mother's subsequent pregnancy: n (CVS and amniocentesis) Mother's second subsequent pregnancy: n (amniocentesis)
8.	Shanske *et al*^39^	*MTND5*	m.13513G>A	89% (M), 80% (Bl)	Mother: n (Bl, U) Mother's subsequent pregnancy: n (amniocentesis) Postpartum analysis of this sister: n (cord blood, Bl) Maternal aunt: n (Bl, U) Maternal grandmother: n (Bl, U)
9.	Marchington *et al*^34^	*MTATP6*	m.9176T>C	99% (in ‘all tissues examined’, not further specified)	Mother: n (Bl, BM, U, 15 oocytes), 40% (2 oocytes together; could not be dissected separately), ≤5% (1 oocyte) Mother's subsequent pregnancy: n (CVS) Postpartum analysis of this sibling: n (16 samples of placenta, cord blood)
10.	Götz *et al*^40^	*MTTS1*	m.7453G>A	100% (M)	Mother: n (Bl) Mother's subsequent pregnancy: n (CVS)
11.	Shanske *et al*^41^	*MTND3*	m.10198C>T	100% (M, heart, liver, brain)	Mother: n (Bl, U, H) Mother's subsequent pregnancy: n (CVS and amniocentesis, also: prenatal fetal muscle biopsy) Postpartum analysis of this sister: n (placenta portion, cord blood, H) Maternal grandmother: n (U)
12.	Nesbitt *et al*,^18^ personal communication	*MTATP6*	m.9176T>C	97% (Bl, M)	Mother: n (Bl, U) Mother's subsequent pregnancy: 98% (CVS) 6 embryos in PGD cycle: n (no pregnancy achieved) Mother's second (spontaneous) subsequent pregnancy: 8% (CVS)
13.	Nesbitt *et al*,^18^ personal communication	*MTND6*	m.14453G>A	65% (M), 39% (F)	Mother: n (M, Bl, U, BM) Mother's subsequent pregnancy: n (CVS)
14.	Unpublished data from Newcastle	*MTND5*	m.13513G>A	81% (M)	Mother: n (Bl) Mother's subsequent pregnancy: n (CVS)

Bl, blood; BM, buccal mucosa; CVS, chorionic villus sampling; F, fibroblasts; H, hair; M, muscle; n, normal (mutation not detected); U, urine.

#### Case 1

Couple 1 was referred for PGD because their son had Leigh syndrome due to the m.8993T>G mutation in the *MTATP6* gene, with 90% mutant load in skeletal muscle. He died at the age of 1. Family history was otherwise negative. His healthy older sister, 6 years of age, was not tested for the mutation. In the mother's blood, hair and muscle the m.8993T>G mutation was absent (detection level <1%). The mutation seeming to have arisen de novo in their son, a low recurrence risk was estimated and PND was offered for reassurance. The couple were surprised because maternal inheritance of the m.8993T>G mutation was assumed, based on the high mutation percentage and severe disease in the affected child, and they were counselled as having a high recurrence risk. Furthermore, they were informed that PND was not an option because of limitations in predicting the phenotype of the offspring and PGD was recommended. The couple opted for PND. The mutation was not detected in chorionic villus DNA and a healthy daughter was born. The child has not been genetically tested post partum, but is still doing well at the age of 11.

#### Case 2

Couple 2 was referred for PGD. Their daughter had a mitochondrial disorder due to oxidative phosphorylation defects (strongly diminished complex I, III and IV activities). She was shown to carry an m.5556G>A mutation in the *MTTW* gene, with >90% heteroplasmy level in skeletal muscle. This mutation had not been described before. Pathogenicity was based on the disruption of the tertiary structure of mt-tRNA^Trp^ by the mutation, and the compatibility of the mutation with the combined deficiency of complexes I, III and IV, which was also demonstrated in *trans*mitochondrial cybrids.^25^ She died when she was 1.5 years old. Family history was negative. The mother's blood, hair, urine and muscle did not show the m.5556G>A mutation (detection level <1%), and accordingly, the mutation was considered de novo in their daughter, further supporting a pathogenic role. The recurrence risk was regarded as low and PND was offered. PGD seemed a less suitable option because of the presumed low recurrence risk and the maternal age (38 years). As in case 1, the couple was surprised since at the referring centre PND was not considered an option because of uncertainties about the representativeness of the prenatal sample for the fetus, about the stability of the mutant load over time, and about the clinical phenotype to be expected. Amniocentesis revealed no mutation. A healthy daughter was born, who is now 4 years old. No mutation analysis was performed post partum.

#### Case 3

The son of couple 3 died at age 7.5 months carrying the m.8969G>A mutation in the *MTATP6* gene with 95% heteroplasmy in blood, fibroblasts and skeletal muscle. The m.8969G>A mutation was not detected in the mother's blood and urine (detection level <1%). The couple opted for PND in a subsequent pregnancy, and amniocentesis showed no mutation. A healthy girl was born, now almost 4 years old. She has not been tested for the mutation post partum.

#### Case 4

Couple 4 was referred to discuss their reproductive options because their son had Leigh syndrome caused by an almost homoplasmic m.8993T>G mutation in the *MTATP6* gene, demonstrated in his blood, fibroblasts and skeletal muscle. He died when he was 5.5 months of age. In the mother's blood, urine and hair, the m.8993T>G mutation was not present (detection level <1%). The mutation therefore appeared de novo in their son, resulting in a low recurrence risk. The boy also had neurofibromatosis type 1 (NF1) caused by a de novo mutation c.2155dupA in the NF1 gene. Since recurrence risks of both the m.8993T>G and the NF1 mutation were low, PND was offered. The subsequent pregnancy ended in a spontaneous miscarriage. The m.8993T>G mutation was not detected in the abortus. Subsequently, a further spontaneous pregnancy was achieved. CVS was performed and the m.8993T>G was not detected in chorionic villi. A healthy daughter was born, who is presently 2 years old. No mutation analysis was performed post partum.

#### Case 5

Couple 5 was referred to us to discuss the possibility of PGD because their son harboured the m.3243A>G *MTTL1* mutation. Mutation loads were 13%, 12%, 17%, 16% and 14% in his blood, skeletal muscle, fibroblasts, urine and buccal mucosa cells, respectively. The mutation was absent in the mother's blood, muscle and buccal mucosa (detection level <1%); the mutation was also absent in the maternal grandmother's blood, urine and buccal mucosa, consistent with a de novo mutation in the index patient and a low recurrence risk of m.3243A>G-related disease. It was doubtful, however, whether the boy's severe, infantile-onset, clinical presentation with hypotonia, feeding problems, psychomotor retardation and intractable epilepsy could be explained by the relatively low mutation load of the m.3243A>G mutation, and just prior to his death at age 3, compound heterozygosity for the POLG mutations c.2740A>C, p.(Thr914Pro) and c.2243G>C, p.(Trp748Ser) was diagnosed, which was consistent with his clinical features of Alpers’ syndrome. Both parents were carriers (father c.2740A>C, p.(Thr914Pro) and mother c.2243G>C, p.(Trp748Ser)), resulting in a recurrence risk of 25% for the recessive *POLG* mutations. The couple preferred PGD to PND. Despite the presumed low recurrence risk for the m.3243A>G mutation, they also requested analysis of the embryos for the m.3243A>G mutation. It was agreed that of the embryos one blastomere was tested for *POLG* and one blastomere for m.3243A>G. Since testing for the m.3243A>G mutation was performed as reassurance, analysis of one blastomere seemed reasonable, although two blastomeres are usually analysed.^14^ In none of the 11 embryos and oocytes the m.3243A>G mutation was detected. One embryo, heterozygous for one of the POLG mutations, was transferred and resulted in a successful pregnancy. The couple did not opt for PND to confirm the PGD result. A healthy son was born. Postpartum DNA analysis was performed in cord blood and confirmed the blastomere genotype and absence of the m.3243A>G mutation.

In the literature, together with one unpublished case from Newcastle, we identified a further 11 prenatal diagnoses, performed in 9 pregnant mothers of a previously identified case of de novo mtDNA disease. These are listed in table 2, together with our own five cases. All but one case (case 12) showed normal prenatal results. One of the mothers (also case 12) additionally underwent one PGD treatment.

## Discussion

Irrespective of the mechanism leading to mtDNA disease in a child, parents may desire to prevent disease in a subsequent child. The reproductive options available to such couples largely depend on the genetic aetiology. Based on data provided from our own clinical experience and from cases published in the literature, we conclude that mtDNA mutations arise de novo in a significant number of cases and that the recurrence risk for apparently de novo mtDNA mutations is low.

A common approach to determine whether an mtDNA mutation occurred de novo is by testing multiple tissues from the mother for the heteroplasmic mtDNA variant. It is, however, remarkable that often only the mother's blood is analysed even when the mutation was not tested or detected in the affected child's blood (see online supplementary table S2). It is well known, at least for certain mtDNA mutations,^26–28^ that mutant load in blood, a rapidly dividing tissue, can decrease over time due to negative selection. This clearly has implications for the reliability of maternal blood in evaluating de novo occurrence of an mtDNA mutation. Therefore, preferably muscle (a postmitotic tissue) should be included in the maternal analysis, which has however the drawback of an invasive procedure. Notably, needle muscle biopsy sampling nowadays offers a less invasive, more rapid alternative to conventional open muscle biopsies, yielding a sufficient muscle amount for DNA analysis. Urine epithelium has been shown to be a reliable non-invasive alternative for the m.3243A>G mutation.^29^ This may also be true for other pathogenic mtDNA point mutations,^30^ although urine mutant load has not been compared with muscle levels for these. Evaluation of tissue distribution is also critical in light of potential selection events in the germline. Negative selection has been suggested for pathogenic tRNA mutations with low blood mutant levels (pointing to detrimental effects in replicating cells), which are less likely to be transmitted and as a consequence occur more often in isolated cases.^31^ Similarly, negative germline selection has been proposed for deleterious heteroplasmic mtDNA mutations and for de novo mutations in a recent study of healthy humans.^32^ These findings implicate that mutations that are absent or (very) low level in the index patient's blood may be more likely to have indeed occurred de novo. This cannot, however, be assumed for individual mutations without careful analysis of the mother. Furthermore, conversely, potential positive selection events in the germline whereby high mutation loads in offspring could result from low maternal levels cannot be excluded and again stress the importance of thorough maternal investigation. In addition to analysis of maternal tissues, testing apparently healthy siblings of the affected child can contribute to the likelihood that a mutation occurred de novo. The method used to assess mtDNA heteroplasmy is of critical importance. Fluorescent last-cycle restriction fragment length polymorphism (RFLP) analysis has a detection level of <1%, whereas Sanger sequencing has a sensitivity of between 5% and 30% to detect different heteroplasmic mutations (unpublished laboratory findings). Even using last-fluorescent RFLP analysis, the absence of a mutation in the mother is obviously not definitive as a mutation load below the detection threshold for the assay in the mother cannot be excluded, neither can the presence of the mutation in her untested tissues, particularly oocytes.

### Proportion of mtDNA point mutations arising de novo in patients with mtDNA disease

Based on the absence of the mtDNA mutation in (mostly multiple) maternal tissue(s), 24.6% of the putative pathogenic mtDNA point mutations in our cohort were de novo, a significant subset of cases. which is in agreement with available data from other centres (see online supplementary table S2).^1^ Since paediatric patients seem over-represented in the de novo subgroup, whereas the majority of the entire cohort are adult patients, the proportion of de novo mutations is likely higher in the paediatric patient population and lower in adults.

De novo mtDNA point mutations manifesting below the threshold required for phenotypic expression are clearly not included in this number. It is, however, important to realise that de novo mutations in asymptomatic individuals could potentially segregate to high levels and thus cause mtDNA diseases in subsequent generations. This mechanism is also illustrated by our cases (grey-coloured cases in online supplementary tables S1 and S2) where a mutation did not occur de novo in the index patient, but in the healthy mother (or another maternal relative). Such carriers with low mtDNA mutation loads themselves will only be identified if they have a clinically affected child.

### Recurrence risk of de novo mtDNA mutation or disease

The recurrence risk of a de novo mtDNA mutation depends on the moment at which the mutation arose. A germline de novo mutation event, which reaches clinical significance, most likely occurs at the lowest point of the bottleneck during oogenesis, when the mtDNA copy number is lowest. The same de novo event is not expected to happen twice and the recurrence risk is therefore negligible. Mutations may also be pre-existent in (some) maternal oocytes, representing gonadal mosaicism and resulting in a potential recurrence risk. Oocyte sampling has been used to further estimate the recurrence risks of mtDNA mutations,^33^ ^34^ although the invasive nature of this procedure may pose ethical questions if no assisted fertility treatment is intended. Also, it is still not guaranteed that the analysed oocytes are representative of the entire oocyte pool. Finally, a de novo mutation may represent a de novo somatic mutation (being by definition not present in the mother) rather than a de novo germline mutation. In the latter scenario, there is no risk of recurrence. Somatic mutations may be present in only one tissue (eg, skeletal muscle); however, also when detected in several tissues, even of different embryonic origin (such as muscle and urinary epithelial cells,^35^ or muscle and hair roots,^36^ respectively), the mtDNA mutation can be somatic, having occurred very early in embryological development. Taken together, it is nigh impossible to distinguish whether an mtDNA mutation occurred de novo somatically or in the germline (and if so, at what point in the germline) in single-disease cases. In practice, all three scenarios should be considered in cases of apparently de novo mtDNA mutations and as such are taken together in empirically established recurrence risks as discussed below.

Our PND/PGD data of subsequent pregnancies obtained from five mothers of patients with de novo mtDNA disease indicate a very low recurrence risk for de novo mtDNA mutations. These results are supported by (un)published data in which PND in subsequent pregnancies following affected children with an apparently de novo mtDNA mutation were reported in nine mothers (table 2^18^ ^34^ ^37–41^). In eight of these, the mtDNA mutation was not detected in the prenatal sample(s). The absence of the mtDNA mutation in 97 siblings of individuals with a presumed de novo mtDNA mutation further adds to the low recurrence risk of these mutations (table 1 and online supplementary table S2). However, in most asymptomatic siblings of an index patient (a low) mtDNA mutation load could not be excluded as they were not tested for the mutation on ethical grounds. Recurrence risk is increased in the case of gonadal mosaicism, which was shown for one of the de novo cases in the literature (case 9, table 2/case 58, online supplementary table S2^34^). The single case where the mutation was present in subsequent pregnancies of the mother (case 12, table 2/case 59, online supplementary table S2^18^) without being detectable in her blood or urine is presumably also an example of gonadal mosaicism. The maternal mutation analysis was repeated using deep NGS analysis, but even with this the mtDNA mutation could not be detected in the mother. It is the only case known so far where three offspring have the same mtDNA mutation that is not detectable in the mother. The pattern of distribution of mutation load in this mother's offspring with very high levels in some (her affected child and one of the prenatal samples), but no mutation in the majority (six embryos), is quite similar to an m.8993T>G carrier with low mutant load we previously described (case 44, online supplementary table S1^14^). Interestingly, the two cases of (presumed) maternal gonadal mosaicism in table 2 concern the same mtDNA mutation, m.9176T>C.^18^ ^34^

The total number of prenatal/preimplantation samples described in this paper is 50, including multiple pregnancies per female (cases 7 and 12, table 2) multiple oocytes and/or embryos per female (cases 5, 9 and 12, table 2) and analysed abortus material (case 4, table 2). In four of these samples, the mtDNA mutation present in the index patient was detected, indicating a recurrence risk of 8% (4/50). Larger numbers of (normal) prenatal diagnoses are presently not available to include in our analysis. However, we do have results in siblings that can be added. Also, 100 siblings of 57 individuals with an apparently de novo mtDNA mutation based on absence of the mutation in the mother (note: families where the mother of such an individual was not analysed, cases 79 and 80 in online supplementary table S2, were not included in this calculation) were tested, both from our own centre and from the literature. In one of these, the mtDNA mutation might be present at low levels in a clinically unaffected sibling (case 108, online supplementary table S2^36^), although recurrence is debatable here since the sibling's mutation load is at the limit of detection. Besides, in the mother only blood was tested, which is also the case in a second recurrence example (case 97, online supplementary table S2^42^). In a third case, the mutation was present (3% mutant load) in the sibling's urine, whereas it was absent in the mother's blood and urine (case 122, online supplementary table S2^43^). Considering both the prenatal/preimplantation data (n=50) and the sibling data (n=100), a recurrence risk of approximately 4% is calculated (5–7/150) in this data set. It is likely that this percentage further decreases when all healthy siblings that were not tested could be included.

Few other reports from family studies potentially describe recurrence, but the data are not unambiguous. These include a case where the similarly affected sibling was not tested for the mtDNA mutation and pathogenicity of the mutation has not been proven (case 93, online supplementary table S2^44^); and two cases where relatives with neurological symptoms were not extensively or not at all tested for the mtDNA mutation, whereby it remained unclear whether these symptoms are related to the familial mtDNA mutation or represent a separate disease (cases 50 and 79, online supplementary table S2^45^ ^46^). Presence of the m.14484T>C mutation in an unaffected sibling, when no mutation was detectable in the mother, was reported in a monozygotic twin (case 136, online supplementary table S2^47^), consistent with no recurrence.

Even if all the cases discussed (with the exception of the monozygotic twin) would actually represent recurrence despite absence of the mutation in the mother, 8 examples in 154 cases (17 from our own centre, 137 from literature) would still support our hypothesis of a low recurrence risk, considering the likely bias that exceptional cases may be more easily reported. Furthermore, it is important to note that in none of the (potential) recurrence cases (including the proven gonadal mosaicism one) postmitotic tissue such as muscle was analysed in the mother. None of these involved the m.3243A>G mutation.

### Reproductive counselling strategy for mtDNA disease

**Figure 2:**
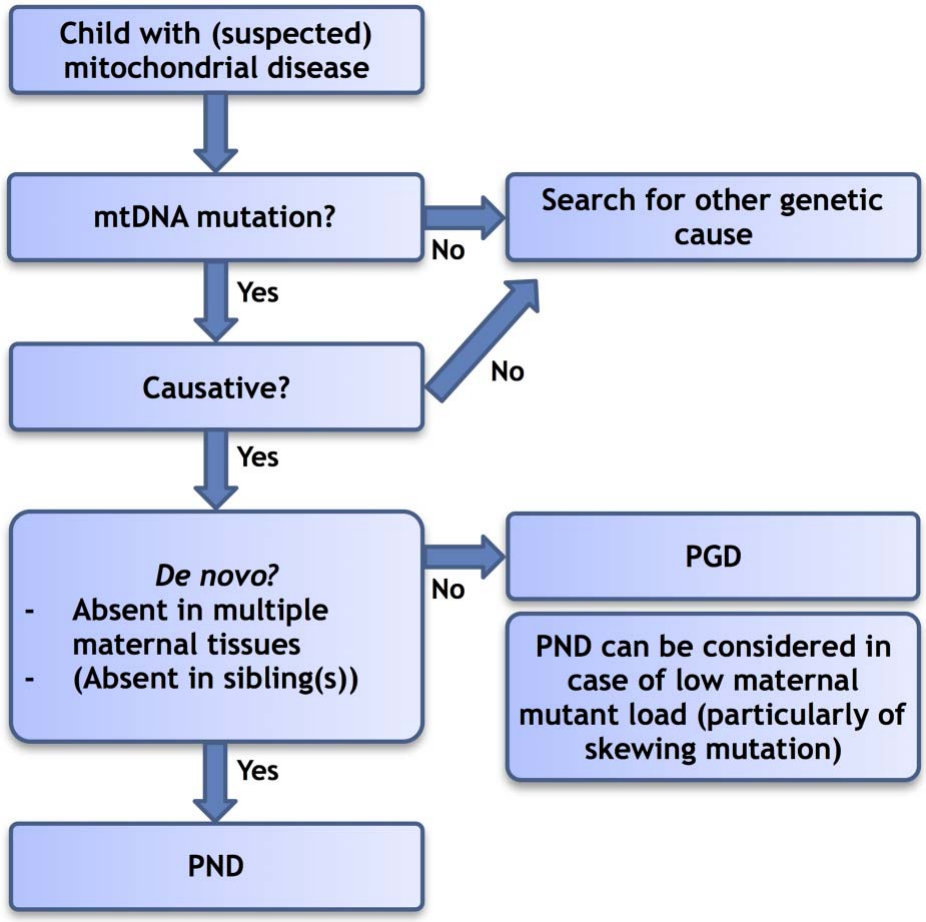
Flow chart: simplified reproductive counselling strategy for mitochondrial (mt)DNA disease. Patient's preferences have not been explicitly included in this algorithm; however, they obviously are an important factor as well. Choices are made on a case-by-case basis. PGD, preimplantation genetic diagnosis; PND, prenatal diagnosis.

When a couple with a child affected by mtDNA disease seeks counselling regarding the likelihood of having subsequent unaffected offspring, an individual risk assessment should be performed, leading to personalised advice (figure 2). The first step is to ascertain the genetic cause of the disease. As is illustrated by case 5, finding a pathogenic mtDNA mutation does not always provide an unequivocal answer and the mtDNA heteroplasmy level and clinical phenotype should be consistent with previous reports of affected individuals. If a causative mtDNA mutation is identified, the next step is to evaluate whether the mutation occurred de novo or was maternally inherited. It is necessary to examine multiple tissues in the mother preferably including a postmitotic tissue such as muscle if possible (needle–biopsy suffices) and being mindful of the tissues anticipated to harbour the mutation at detectable levels. For the m.3243A>G mutation, urine can be considered a reliable alternative for muscle, but for other mutations this correlation has not been validated. A quantitative method with low detection level (<1–2%), such as fluorescent RFLP, pyrosequencing or NGS, should be used. Testing of unaffected siblings of the affected child should be considered but may be deemed unacceptable on ethical grounds. As it will be impossible to fully exclude a mutation in the mother, partly due to technical reasons and the availability of tissues or oocytes (note: oocyte sampling might be considered), couples should be aware of a small residual risk. Based on our current calculation, this risk may be ∼4%, although the actual risk is likely to be lower. If a couple wishes reproductive genetic testing, PND is the preferred option given the high likelihood that the mutation will not be present in the fetus. PND is also a fair option for carriers with low mutation load (eg, <10%) of familial mutations, particularly for an mtDNA mutation that manifests skewed segregation, and for single, large-scale mtDNA deletions, the latter both for healthy mothers of an affected child and for affected women themselves. Although amniocentesis seems to be the preferred method for some mtDNA mutations,^48^ one can discuss the relevance of this in situations where no mutation is to be expected at all. CVS, on the other hand, has the advantage that results can be obtained earlier in the pregnancy. In most familial mtDNA mutations, with a high or unpredictable recurrence risk in offspring, and given the potential difficulties of interpreting PND results, PGD is currently the best reproductive option.^14^ Decisions on reproductive options naturally also depend on personal considerations of the individual couple; providing all the available options and information is the key to being able to make a well-informed decision.
