# Supplementary material for: De novo mtDNA point mutations are common and have a low recurrence risk
Source: J Med Genet. 2016 Jul 22;54(2):73–83. doi: 10.1136/jmedgenet-2016-103876 (PMC5502310; doi:10.1136/jmedgenet-2016-103876)
Supplement: supplementary tables [file jmedgenet-2016-103876supp001.pdf]

Supplementary Table S1 (Likely) pathogenic mtDNA mutations identified in our diagnostic laboratory (Maastricht), not (established to be) *de novo* in the index patients. Mutations are listed according to maternal or unknown inheritance, and according to nucleotide position.

	Reference	Family no.	Gene	Mutation	Mutation load(s) in tested tissue(s) of index patient	Mutation load(s) in tested tissues of (maternal) relative(s)	Index patient's age at investigation
**Maternally inherited cases**							
1.	-	1684	*MTTL1*	m.3243A>G	27% (Bl), 32% (M), 73% (heart)	Sister: 15-16% (Bl), 38-39% (U)<br>Brother: 6% (Bl), 51% (U)<br>Daughter of maternal aunt: 2-3% (Bl)<br>This daughter's daughter: n (Bl, M)<br>Other daughter of maternal aunt: 5% (Bl), 27% (M)<br>Maternal uncle: 12% (Bl)	31
2.	-	2155	*MTTL1*	m.3243A>G	32% (Bl), 77% (F), 61% (M)	Maternal aunt: 65% (M), 64% (F)	36
3.	-	2211	*MTTL1*	m.3243A>G	53% (M), 5% (Bl)	Mother: n (Bl), 9% (M)<br>Daughter of maternal aunt: n (Bl)	35
4.	-	2876	*MTTL1*	m.3243A>G	23% (M), 18% (Bl)	Mother: 5% (Bl)<br>Sister: n (Bl, U)	8
5.	-	4547	*MTTL1*	m.3243A>G	8-13% (Bl), 12% (H), 19% (M)	Mother: 2% (Bl), 5% (F), 7% (M)	16
6.	-	4907	*MTTL1*	m.3243A>G	13-19% (Bl)	Son of maternal aunt: 9% (Bl)<br>Daughter of maternal aunt: n (Bl)	44
7.	Sallevelt et al. 2013 [14]	7237	*MTTL1*	m.3243A>G	35% (Bl), 40% (H)	Mother: 9% (Bl), 8% (H), 15% (M)<br>Sister: 25% (Bl), 10% (H), 28% (M)<br>Sister: 23% (Bl), 9% (H), 28% (M)	26
8.	-	7578	*MTTL1*	m.3243A>G	14% (Bl), 33% (H), 77% (F), 73% (M)	Brother: 12% (Bl), 52% (H), 40% (F), 60% (M)	33
9.	-	7892	*MTTL1*	m.3243A>G	9% (Bl)	Daughter: 25% (Bl)<br>Sister's daughter: n (Bl, H, M), 1% (BM), 3% (U),	60
10.	-	8037	*MTTL1*	m.3243A>G	77% (M), 30% (unknown tissue)	Mother: 7-9% (Bl), 55% (M), 16% (U)<br>Sister: 30% (Bl), 74% (M)<br>Sister: 16% (Bl), 42% (M), 38% (U)	27

						Maternal aunt: n (Bl, BM, M, H, U)<br>Maternal uncle: n (Bl, U)<br>Maternal grandmother: n (Bl), <5% (U)	
11.	-	8515	*MTTL1*	m.3243A>G	41% (Bl), 58% (H), 59% (F), 63% (M)	Mother: 5% (Bl), 31% (M)	26
12.	-	11111	*MTTL1*	m.3243A>G	20% (Bl), 83% (M)	Brother: 7% (Bl)	51
13.	-	11368	*MTTL1*	m.3243A>G	7% (Bl), 25% (U)	Sister: 15% (Bl)	61
14.	-	13412	*MTTL1*	m.3243A>G	28% (Bl)	Mother: 8% (Bl)<br>Maternal aunt: n (Bl)<br>Maternal aunt: n (Bl)	35
15.	-	14508	*MTTL1*	m.3243A>G	13% (Bl), 74% (U)	Mother: 2% (Bl), 10% (U)<br>Half-sister (same mother): n (Bl, U)	46
16.	-	14890	*MTTL1*	m.3243A>G	60% (Bl)	Mother: 10% (Bl), 53% (U), 13% (H)<br>Brother: 35% (Bl), 40% (H), 81% (U)<br>Sister: 1% (Bl, U, H)	14
17.	-	15357	*MTTL1*	m.3243A>G	17% (Bl)	Brother: 15% (Bl)	51
18.	-	16104/19160	*MTTL1*	m.3243A>G	29% (Bl)	Sister: 15% (Bl), 24% (H), 30% (U)	38
19.	Sallevelt et al. 2013 [14]	16221	*MTTL1*	m.3243A>G	40% (Bl)	Mother: 40% (Bl)<br>Sister: 27% (Bl), 29% (H), 59% (U)<br>Maternal aunt: 8% (Bl), 12% (H), 29% (U)<br>Maternal aunt: 5-10% (Bl)	32
20.	-	16443	*MTTL1*	m.3243A>G	27% (Bl)	Sister: 14% (Bl)<br>Maternal aunt: n (Bl, U)	41
21.	-	18248	*MTTL1*	m.3243A>G	69% (Bl)	Mother: 17% (Bl)	9
22.	-	18350	*MTTL1*	m.3243A>G	28% (Bl), 86% (U), 85% (F), 73% (M)	Mother: 2% (Bl), 8% (saliva)<br>Sister: 2% (Bl)<br>This sister's son: 44% (Bl)<br>Sister: n (Bl, U)	35
23.	-	19336	*MTTL1*	m.3243A>G	16-19% (Bl)	Sister: 32% (Bl)	37
24.	-	19533	*MTTL1*	m.3243A>G	9% (Bl), 20% (U)	Sister: 10% (Bl), 35% (U), 77% (M)<br>Sister's daughter: 23% (Bl), 70% (M)	52
25.	-	19885	*MTTL1*	m.3243A>G	15% (Bl), 75% (U)	Sister: 3% (Bl)<br>Brother: 9% (Bl)<br>Brother: 9% (Bl)	49

26.	-	20108	*MTTL1*	m.3243A>G	11% (Bl)	Sister: 9% (Bl), 29% (U)<br>Sister: 19% (Bl), 35% (U)<br>This sister's daughter: 39% (Bl), 83% (U)	52
27.	-	21101	*MTTL1*	m.3243A>G	51% (Bl), 93% (M)	Mother: 17% (Bl), 72% (M)<br>Sister: 25% (Bl), 57% (M)<br>Sister: 23% (Bl), 40% (M)<br>Maternal aunt: 9% (Bl)	20
28.	-	21200	*MTTL1*	m.3243A>G	14% (Bl), 85% (U)	Sister: 28% (Bl), 57% (U)<br>Brother: 32% (Bl), 92% (U)<br>Sister: 17% (Bl), 43% (U)<br>This sister's daughter: n (Bl, U)	59
29.	-	23232	*MTTL1*	m.3243A>G	? (not tested in our laboratory)	Sister: mutation detected (not tested in our laboratory)<br>Sister: mutation detected (not tested in our laboratory)<br>Daughter: 19% (Bl), 26% (H), 38% (U)	56
30.	-	23466	*MTTL1*	m.3243A>G	? (not tested in our laboratory)	Mother: mutation detected (not tested in our laboratory)<br>Maternal aunt: mutation detected (not tested in our laboratory)<br>Maternal aunt: 38% (Bl),59% (U)<br>Maternal grandmother: mutation detected (not tested in our laboratory)	Exact age unknown (child)
31.	-	8799	*MTTL1*	m.3243A>G	16% (Bl), 53% (U)	Mother: 10% (Bl), 68% (U)<br>Maternal uncle: 4% (Bl), 70% (U)<br>Maternal uncle: 14% (Bl), 81% (U)<br>Maternal aunt: 2% (Bl), 19% (U)<br>Maternal aunt: 9% (Bl), 57% (U)<br>Maternal aunt: n (Bl), 37% (U)<br>Several children of maternal uncles/aunts also tested and mutation detected	44
32.	-	26435	*MTTL1*	m.3243A>G	32% (Bl), 58% (U)	Mother: n (Bl), 20% (U)	36
33.		2242	*MTTL1*	m.3271T>C	100% (Bl)	Mother: 73% (Bl)<br>Brother: 100% (M)	7
34.	-	15426	*MTTL1*	m.3291T>C	56% (Bl), 94% (M)	Mother: 26% (Bl)	7

						Brother: heteroplasmic, no exact % (Bl)<br>Maternal grandmother: 3% (Bl)	
35.	Van denBosch et al. 2004 [50]	3394	*MTTL1*	m.3302A>G	62% (Bl), 60% (F), 80% (H), 96% (F)	Mother: 76% (M), 33% (Bl), 44-84% (F), 58% (H), 67% (breast tissue)<br>Sister: 23% (H), 26% (Bl)	34
36.	-	22190	*MTTL1*	m.3303C>T	99% (Bl), 100% (M)	Mother: 82% (Bl), 85% (H), 76% (saliva), 84% (U)<br>Mother's subsequent pregnancy: 38% (amniocentesis)	0
37.	Spruijt et al. 2007 [51]	7141	*MTND1*	m.3697G>A	56% (Bl), 97% (M)	Mother: 19% (Bl)<br>Brother: 88% (Bl), >97% (M)	36
38.	-	10947	*MTND1*	m.3890G>A	88% (M)	Mother: 2% (M), 3% (Bl)<br>Sister: 1% (M), 1% (Bl)	5
39.	-	1829	*MTTK*	m.8344A>G	53% (Bl)	Brother: 17% (Bl)<br>Brother: 31% (Bl), 34% (M)<br>Daughter of maternal aunt: n (Bl)<br>Daughter of other maternal aunt: 83% (Bl)<br>Daughter of this cousin: 85% (Bl)	36
40.	-	4043	*MTTK*	m.8344A>G	86% (not tested in our laboratory)	Brother: 74% (not in our laboratory)<br>Brother: 32% (not in our laboratory)<br>Mother: 69% (not in our laboratory)<br>Maternal aunt: 40% (Bl), 48% (U), 41% (H), 26% (M)<br>Daughter of this aunt: 37% (Bl), 35% (U), 16% (M)<br>Son of this aunt: 15% (Bl), 14% (U), 11% (H)<br>Daughter of this aunt: 32% (Bl), 35% (U), 15% (H)<br>Daughter of this aunt: 92% (Bl), 91% (U), 88% (H), 88% (M)<br>Maternal uncle: 83% (M)<br>Maternal grandmother: 65% (M)<br>Sister of maternal grandmother: 38% (Bl)	10

						Son of this sister: 11% (Bl)	
41.	-	9849	*MTTK*	m.8363G>A	25% (Bl), 90% (M), 21% (U)	Mother: 21% (Bl), 31% (M), 41% (U)<br>Sister: 25% (Bl), 51% (M), 16% (U)<br>Maternal aunt: n (Bl, U, F, M)<br>Maternal aunt: 36% (Bl), 2% (M, U)<br>Daughter of this aunt: 45% (Bl), 93% (M), 40% (F)<br>Daughter of this aunt: 56% (Bl), 63% (U), 2% (F, M)<br>Maternal aunt: 21% (Bl), 1% (F)<br>Maternal uncle: 20% (Bl)<br>Daughter of deceased (not tested) maternal aunt: 34% (Bl), 41% (M)<br>Son of deceased (not tested) maternal aunt: 8% (Bl), 46% (M)	38
42.	-	18198	*MTTK*	m.8363G>A	67% (Bl), 98% (M)	Sister: 15% (Bl)	32
43.	-	21416	*MTTK*	m.8363G>A	? (not tested in our laboratory)	Mother: 48% (Bl)	? (probably adult age)
44.	Sallevelt et al. 2013 [14]	14125	*MTATP6*	m.8993T>G	100% (M), 92% (Bl)	Mother: 4% (Bl), 3% (H), 5% (U)<br>Maternal grandmother: n (Bl, H, U)<br>Maternal aunt: n (Bl, H, U)<br>Maternal great-grandmother: n (Bl, H, U)	0
45.	-	20026	*MTATP6*	m.8993T>G	93% (Bl)	Mother: 12% (Bl)	6
46.	Jacobs et al. 2005 [17]	2819	*MTATP6*	m.9176T>C	93% (M), 90% (F)	Mother: 25% (Bl), 43% (H), 7% (F)<br>Brother: 93% (M), 94% (H)<br>Sister: 92% (Bl), 93% (H)<br>Maternal aunt: 23% (Bl), 43% (H)<br>Daughter of maternal aunt: 55% (Bl), 57% (H)<br>This daughter’s pregnancy: 87% (CVS), 88% (amniocentesis)	9
47.	-	28475	*MTATP6*	m.9176T>C	100% (not tested in our laboratory)	Mother: heteroplasmic mutation detected (not tested in our laboratory)<br>Three sisters: heteroplasmic mutation detected (not tested in our laboratory)	40

						Daughter of one of the sisters: 90% (Bl), 92% (U), 94% (H)<br>Other daughter of this sister: 99-100% (not tested in our laboratory)	
48.	Blok et al. 2007 [49]	7964	*MTND5*	m.13042G>A	77% (Bl), 84% (M), 86% (F)	Mother: 11% (Bl), 25% (H)<br>Maternal grandmother: <2% (Bl), 2-4% (M)	2
49.	-	15113	*MTND5*	m.13513G>A	80% (M, not tested in our laboratory)	Mother: 2% (U), n (Bl, H)	0
50.	-	9572	*MTND6*	m.14484T>C	~100% (M)	Mother: ~100% (Bl)	1
51.	-	22016	*MTTE*	m.14674T>C	~100% (Bl, M)	Mother: ~100% (Bl)	11
52.	-	4764	*MTTT*	m.15908T>C	100% (Bl, M)	Brother: 100% (Bl)	29
**Unknown whether *de novo* or maternally inherited**							
53.	-	21724	*MTRNR1*	m.1494C>T	~100% (M)	-	1
54.	-	18893	*MTRNR1*	m.1555A>G	~100% (Bl, M)	-	52
55.	-	22065	*MTRNR1*	m.1555A>G	~100% (Bl)	-	40
56.	-	2899	*MTTL1*	m.3243A>G	72% (F), 85% (H), 94% (M)	-	35
57.	-	3552	*MTTL1*	m.3243A>G	50% (M), 1% (Bl)	Son: 16% (Bl)	51
58.	-	5761	*MTTL1*	m.3243A>G	2% (M), n (Bl)	-	11
59.	-	7943	*MTTL1*	m.3243A>G	4% (Bl)	-	59
60.	Sallevelt et al. 2013 [14]	9433	*MTTL1*	m.3243A>G	6-8% (Bl)	Daughter: 13% (Bl), 26% (H), 55% (U) (NB based on family history a familial mutation is suspected)	56
61.	-	10228	*MTTL1*	m.3243A>G	32% (Bl)	-	40
62.	-	12426	*MTTL1*	m.3243A>G	50% (Bl)	-	54
63.	-	12671	*MTTL1*	m.3243A>G	25% (Bl), 30% (U)	-	30
64.	-	12786	*MTTL1*	m.3243A>G	50% (Bl)	-	38
65.	-	12984	*MTTL1*	m.3243A>G	5% (Bl), 10% (M)	Son: n (Bl, U, M)	46
66.	-	14806	*MTTL1*	m.3243A>G	22% (Bl), 43% (H), 85% (U)	-	35
67.	-	16023	*MTTL1*	m.3243A>G	65% (Bl)	-	9
68.	-	16744	*MTTL1*	m.3243A>G	27% (Bl)	-	35

69.	-	17787	*MTTL1*	m.3243A>G	12% (Bl)	-	63
70.	-	18059	*MTTL1*	m.3243A>G	37% (Bl), 45% (H), 60% (U)	-	30
71.	-	19162	*MTTL1*	m.3243A>G	% unknown (not tested in our laboratory)	Daughter: 16% (Bl), 29% (H), 28% (U)	Between 38 and 49
72.	-	20667	*MTTL1*	m.3243A>G	16% (Bl), 62% (M), 54% (F)	-	59
73.	-	21909	*MTTL1*	m.3243A>G	25% (Bl), 76% (M)	-	27
74.	-	23847	*MTTL1*	m.3243A>G	30% (Bl, not tested in our laboratory)	Daughter: 45% (Bl), 52% (H), 77% (U)	58
75.	-	24390	*MTTL1*	m.3243A>G	27% (Bl), 90% (U)	-	38
76.	-	24556	*MTTL1*	m.3243A>G	18% (Bl)	-	50
77.	-	26538	*MTTL1*	m.3243A>G	18% (Bl)	-	55
78.	-	26586	*MTTL1*	m.3243A>G	11% (Bl)	-	70
79.	-	7403	*MTTL1*	m.3243A>T	34% (M)	-	57
80.	-	4207	*MTTL1*	m.3260A>G	91% (M)	-	46
81.	-	13686	*MTTK*	m.8363G>A	80% (Bl)	- (NB based on family history a familial mutation is suspected)	45
82.	-	7624	*MTTK*	m.8363G>A	97% (M)	-	43
83.	-	20850	*MTATP6*	m.9185T>C	~100% (Bl, M)	-	57
84.	-	18920	*MTND3*	m.10158T>C	39% (Bl), 97% (M)	-	55
85.	-	17242	*MTND4*	m.11757A>G	Heteroplasmic, no exact % due to semi-quantitative method (Bl, M)	-	1
86.	-	16849	*MTTL2*	m.12297T>C	~100% (Bl, M)	-	43
87.	-	12777	*MTND5*	m.13042G>A	99% (tissue unknown, sent from abroad)	- (NB based on family history a familial mutation is suspected)	46
88.	-	11478	*MTND5*	m.13514A>G	12% (Bl)	-	37

Grey-colored cases: not de novo in index patient, but in other (maternal) relative.

Light grey-colored cases: possibly de novo in other (maternal) relative

M: muscle Bl: blood U: Urine F: fibroblasts H: hair BM: buccal mucosa CVS: chorionic villus sampling
n: normal (mutation not detected)
~ refers to approximate mutation percentage (determined by semi-quantitative method: sequence analysis)

Supplementary Table S2 Presumably *de novo* mtDNA mutations in the literature.
Mutations are listed according to whether maternal and/or sibling's tissues were tested and according to nucleotide position.

	Reference	Gene	Mutation	Mutation load(s) in tested tissue(s) of index patient	Mutation load(s) in tested tissues of maternal relative(s)
**One maternal tissue tested, no siblings tested** [a]					
1.	Hanna et al. 1998 [52]	*MTTF*	m.583G>A	58% (M), n (Bl, F)	Mother: n (Bl)
2.	Darin et al. 2006 [53]	*MTTF*	m.583G>A	79% (M), n (Bl, F)	Mother: n (Bl)
3.	Shoffner et al. 1995 [54]	*MTTL1*	m.3271delT	>95% (M)	Mother: n (Bl)
4.	Ugalde et al. 2007 [55]	*MTND2*	m.4681T>C	>95% (M, F, Bl)	Mother: n (Bl)
5.	Granadillo et al. 2014 [56]	*MTTW*	m.5540G>A	26% (Bl), 51% (M)	Mother: n (Bl)
6.	Anitori et al. 2005 [46]	*MTTW*	m.5543T>C	95% (M), n (Bl, F, 21 hair roots), trace (BM, 1 hair root), 4% (1 hair root), 7% (1 hair root)	Mother: n (Bl)
7.	Moraes et al. 1993 [57]	*MTTN*	m.5703G>A	69% (M), 4% (Bl), 6% (F)	Mother: n (Bl)<br>Two maternal aunts: n (Bl)
8.	Valente et al. 2009 [58]	*MTCO1*	m.6698delA	70% (M), 15% (U), n (Mb)	Mother: n (Bl)
9.	Kollberg et al. 2005 [59]	*MTCO1*	m.6708G>A	81-89% (M), n (Mb, F, H, Bl)	Mother: n (Bl)
10.	Gattermann et al. 1997 [60]	*MTCO1*	m.6721T>C	50% (bone marrow), 50% (Bl), n (lymphocytes, BM), 50% (granulocytes), 20% (platelets)	Mother: n (Bl)<br>Two daughters: n (Bl)
11.	Götz et al. 2012 [40]	*MTTS1*	m.7453G>A	100% (M)	Mother: n (Bl)<br>Mother's subsequent pregnancy: n (CVS)
12.	Seneca et al. 2005 [61]	*MTTD*	m.7526A>G	Nearly100% (M), <3% (Bl, F)	Mother: n (M)
13.	Rahman et al. 1999 [62]	*MTCO2*	m.7671T>A	90% (M), 4.5-6% (Bl)	Mother: n (Bl)
14.	McFarland et al. 2004 [63]	*MTCO2*	m.7989T>C	>90% (M, COX-deficient fibers), <52% (M, COX-positive fibers)	Mother: n (Bl)
15.	De Meirleir et al. 1995 [64]	*MTATP6*	m.8851T>C	100% (tissue?), 97% (F)	Mother: 85% (tissue?)<br>Maternal grandmother: n (tissue?)
16.	Duno et al. 2013 [65]	*MTATP6*	m.8989G>C	33% (Bl), 92% (M), 94% (U), 88% (BM)	Mother: n (Bl)
17.	Santorelli et al. 1993 [66]	*MTATP6*	m.8993T>G	90% (M, F), 84% (muscle cultures)	Mother: 41% (Bl)<br>Maternal grandmother: n (Bl)
18.	Pastores et al. 1994 [67]	*MTATP6*	m.8993T>G	90% (M, F)	Mother: 38% (Bl)<br>Maternal grandmother: n (Bl)
19.	Uziel et al. 1997 [68]	*MTATP6*	m.8993T>G	95% (M)	Mother: 65% (Bl)<br>Maternal grandmother: n (Bl)
20.	Uziel et al. 1997 [68]	*MTATP6*	m.8993T>G	83% (Bl)	Mother: 30% (Bl)

					Maternal grandmother: n (Bl)
21.	Uziel et al. 1997 [68]	*MTATP6*	m.8993T>G	87% (M)	Mother: n (Bl)
22.	Uziel et al. 1997 [68]	*MTATP6*	m.8993T>G	95% (Bl)	Mother: n (Bl)<br>Maternal grandmother: n (Bl)
23.	White et al. 1999 [42]	*MTATP6*	m.8993T>G	97% (F), 100% (M, heart), 98% (liver, kidney)	Mother: n (Bl)
24.	White et al. 1999 [42]	*MTATP6*	m.8993T>G	97% (F), 100% (M, heart), 98% (liver, kidney)	Mother: n (Bl)
25.	White et al. 1999 [42]	*MTATP6*	m.8993T>G	97% (F), 100% (M, heart), 98% (liver, kidney)	Mother: n (Bl)
26.	White et al. 1999 [42]	*MTATP6*	m.8993T>G	100% (M, heart, liver)	Mother: n (Bl)
27.	White et al. 1999 [42]	*MTATP6*	m.8993T>G	76% (F)	Mother: n (Bl)
28.	White et al. 1999 [42]	*MTATP6*	m.8993T>G	94% (F), 95% (M), 96% (heart), 82% (lung, spleen), 90% (liver), 93% (testis), 88% (thyroid)	Mother: 51% (Bl, F)<br>Bother: 89% (Bl), 91% (F)<br>Brother: 86% (Bl, thymus), 91% (heart, liver, thyroid), 89% (lung), 87% (spleen), 92% (cerebellum), 93% (kidney), 85% (meninges)<br>Maternal grandmother: 17% (Bl)<br>Daughter of maternal grandmother's sister: n (Bl)<br>Maternal great-grandmother: n (Bl)
29.	Playán et al. 2002 [69]	*MTATP6*	m.8993T>G	>95% (tissue unknown, only abstract available)	Mother: n (Bl)<br>Maternal grandmother: n (Bl)<br>Maternal aunt: n (Bl)
30.	Steffann et al. 2007 [38]	*MTATP6*	m.8993T>G	90% (Bl)	Mother: n (Bl)<br>Mother's subsequent pregnancy: n (CVS and amniocentesis)<br>Mother's second subsequent pregnancy: n (amniocentesis)
31.	Keightley et al. 1996 [70]	*MTCO3*	m.9487del15	92% (M), 0.7% (Bl), n (F), 0.5% (EBV-transformed lymphocytes)	Mother: n (Bl)
32.	Lebon et al. 2003 [37]	*MTND3*	m.10158T>C	85% (M)	Mother: n (Bl)<br>Mother's subsequent pregnancy: n (CVS and amniocentesis)
33.	Lebon et al. 2003 [37]	*MTND3*	m.10191T>C	90% (M)	Mother: n (Bl)

34.	Lebon et al. 2003 [37]	*MTND3*	m.10191T>C	80% (M)	Mother: n (Bl)
35.	McFarland et al. 2004 [71]	*MTND3*	m.10191T>C	>98% (F, liver)	Mother: n (Bl)
36.	Bannwarth et al. 2008 [72]	*MTND3*	m.10191T>C	68% (Bl), 69% (M)	Mother: n (Bl)<br>Maternal aunt: n (Bl)
37.	Werner et al. 2009 [73]	*MTND3*	m.10191T>C	73% (M), 8-15% (Bl), 13% (F)	Mother: n (Bl)
38.	Komaki et al. 2003 [74]	*MTND4*	m.11777C>A	83% (M), 40% (Bl), 78% (Mb), 57% (F)	Mother: n (Bl)
39.	Zhadanov et al. 2007 [75]	*MTND5*	m.12706T>C	~50% (Bl)	Mother: n (Bl)
40.	Lebon et al. 2003 [37]	*MTND5*	m.12706T>C	60% (M)	Mother: n (Bl)
41.	Valente et al. 2009 [58]	*MTND5*	m.13094T>C	50% (M), 40% (Bl), 30% (F)	Mother: n (Bl)<br>Maternal aunt: n (Bl)<br>Two maternal uncles: n (Bl)
42.	Lebon et al. 2003 [37]	*MTND5*	m.13514A>G	90% (heart)	Mother: n (Bl)
43.	Kirby et al. 2000 [76]	*MTND6*	m.14459G>A	99% (F), 95% (M), >99% (Bl), 98% (liver)	Mother: n (Bl)<br>Maternal grandmother: n (Bl)
44.	Legros et al. 2001 [20]	*MTCYB*	m.15150G>A	60% (M), n (F, Bl, Mb, lymphoblastoid cells)	Mother: n (Bl)
45.	Valnot et al. 1999 [77]	*MTCYB*	m.15243G>A	100% (F), 90% (heart)	Mother: n (Bl)
46.	Wibrand et al. 2001 [78]	*MTCYB*	m.15579A>G	88% (M), 15% (Bl)	Mother: n (Bl)
47.	Lamantea et al. 2002 [79]	*MTCYB*	m.15800C>T	45% (M), n (Bl, F, BM, H, vesical mucosa)	Mother: n (Bl)
**Multiple maternal tissues tested, no sibling(s) tested**[₳]					
48.	Valente et al. 2009 [58]	*MTND1*	m.3688G>A	100% (M, Bl, F)	Mother: n (Bl, F)
49.	Schaller et al. 2011 [80]	*MTTI*	m.4308G>A	47% (M), n (Bl)	Mother: n (M, Bl)
50.	Baríc et al. 2013 [45]	*MTTW*	m.5522G>A	76% (M), 11% (U), 5% (Bl)	Mother: n (Bl, U). Mother has abnormal EMG, elevated CK and myoglobine. Refused muscle biopsy.
51.	Blakely et al. 2007 [81]	*MTTK*	m.8328G>A	82% (M), n (Bl, U, BM)	Mother: n (Bl, U, BM)
52.	Jeppesen et al. 2014 [82]	*MTTK*	m.8340G>A	53% (M), 1% (Bl), 7% (U), 3% (BM)	Mother: n (M, Bl, U, BM)<br>Maternal aunt: n (M, Bl, U, BM)<br>Two children of other maternal aunt: n (M, Bl, U, BM)<br>Son: n (U, BM)
53.	Biancheri et al. 2010 [83]	*MTTK*	m.8344A>G	92% (Bl), 88% (M), 87% (F)	Mother: n (Bl, U)

54.	Burrage et al. 2014 [84]	*MTATP6*	m.8969G>A	89%/96% (Bl), 80%/88% (M), 85% (F)	Mother: n (Bl, U, H)
55.	Seller et al. 1997 [85]	*MTATP6*	m.8993T>G	>95% (F)	Mother: n (Bl, BM)
56.	White et al. 1999 [42]	*MTATP6*	m.8993T>G	96% (Bl), 100% (M, heart, lung, liver, spleen, frontal lobe, cerebellum, kidney)	Mother: n (Bl, F)
57.	De Praeter et al. 2015 [86]	*MTATP6*	m.8993T>G	98% (Bl), 100% (U)	Mother: n (Bl, U, M)
58.	Marchington et al. 2010 [34]	*MTATP6*	m.9176T>C	99% ("all tissues examined", not further specified)	Mother: n (Bl, BM, U, 15 oocytes), 40% (2 oocytes together; could not be dissected apart), ≤5% (1 oocyte)<br>Mother's subsequent pregnancy: n (CVS)<br>Postpartum analysis of this sibling: n (16 samples of placenta, cord blood)
59.	Nesbitt et al. 2014 [18], personal communication	*MTATP6*	m.9176T>C	97% (Bl, M)	Mother: n (Bl, U)<br>Mother's subsequent pregnancy: 98% (CVS)<br>6 embryos in PGD cycle: n (no pregnancy achieved)<br>Mother's second (spontaneous) subsequent pregnancy: 8% (CVS)
60.	Hanna et al. 1998 [52]	*MTCO3*	m.9952G>A	57% (M), n (F, Mb, Bl)	Mother: n (M, Mb, Bl)<br>Two sons: n (M, Mb, Bl)
61.	Leng et al. 2015 [87]	*MTND3*	m.10197G>A	85% (M), 61% (Bl), 76% (U)	Mother: n (Bl, U)
62.	Shanske et al. 2013 [41]	*MTND3*	m.10198C>T	100% (M, heart, liver, brain)	Mother: n (Bl, U, H)<br>Mother's subsequent pregnancy: n (CVS and amniocentesis, also: prenatal fetal muscle biopsy)<br>Postpartum analysis of this sister: n (placenta portion, cord blood, H)<br>Maternal grandmother: n (U)
63.	Nesbitt et al. 2012 [88]	*MTND3*	m.10191T>C	74% (M), 65% (Bl)	Mother: n (Bl, U)
64.	Nesbitt et al. 2012 [88]	*MTND3*	m.10191T>C	87% (M), 64% (F), 87% (liver)	Mother: n (Bl, U)
65.	Roos et al. 2013 [89]	*MTTR*	m.10437G>A	90% (M), 82% (U), n (F, H, BM, Bl)	Mother: n (Bl, H, BM, U)
66.	Wong et al. 2006 [90]	*MTTS2*	m.12207G>A	92% (M), 0.3% (H)	Mother: n (Bl, H)
67.	Karadimas et al. 2002 [91]	*MTTL2*	m.12315G>A	62% (M), 17% (Bl), n (H, U, BM, F)	Mother: n (Bl, U, H)
68.	Alston et al. 2010 [92]	*MTND5*	m.12425delA	85% (M), 14% (Bl), 19% (U), 22% (BM)	Mother: n (Bl, U, BM)
69.	Slawek et al. 2012 [93]	*MTND5*	m.13042A>G	69% (M), 30% (Bl), unspecified% (H),	Mother: n (Bl, H, U)

				unspecified% (U) (see figure in the report)	
70.	Shanske et al. 2008 [39]	*MTND5*	m.13513G>A	89% (M), 80% (Bl)	Mother: n (Bl, U)<br>Mother's subsequent pregnancy: n (amniocentesis)<br>Postpartum analysis of this sister: n (cord blood, Bl)<br>Maternal aunt: n (Bl, U)<br>Maternal grandmother: n (Bl, U)
71.	Nesbitt et al. 2014 [18], personal communication	*MTND6*	m.14453G>A	65% (M), 39% (F)	Mother: n (Bl, U, BM)<br>Mother's subsequent pregnancy: n (CVS)
72.	Leshinsky-Silver et al 2011 [94]	*MTND6*	m.14487T>C	85% (M), 25% (Bl)	Mother: n (Bl, U)
73.	Lax et al. 2013 [95]	*MTTE*	m.14685G>A	24% (U), 44% (M), 7% (heart)	Mother: n (U, Bl)<br>Daughter: n (U, Bl)
74.	Mayr et al. 2006 [96]	*MTTE*	m.14739G>A	72% (M), 38% (U), 31% (Bl), 29% (F)	Mother: n (Bl, U)<br>Maternal grandmother: n (U)<br>Four maternal aunts: n (U)
75.	De Coo et al. 1999 [97]	*MTCYB*	m.14787del4	>95% (M), ~60% (Bl, H, BM, F)	Mother: n (Bl, H, BM)
76.	Emmanuele et al. 2013 [98]	*MTCYB*	m.14864T>C	39% (M), 32% (Bl), 57% (U), 42% (F)	Mother: n (Bl, U)
77.	Fragaki et al. 2009 [99]	*MTCYB*	m.15635T>C	100% (M, Bl, F, liver)	Mother: n (Bl, U, BM)
78.	Mancuso et al. 2003 [35]	*MTCYB*	m.15761G>A	73% (M), <1% (U), n (Bl)	Mother: n (M, U, Bl)
**No maternal tissue tested, sibling(s) tested** [Ⱥ]					
79.	Anitori et al. 2005 [46]	*MTTL1*	m.3287C>A	50% (M), 8% (Bl), 6% (F), 23% (BM), trace to 33%, mean 13,4% (10 hair roots), n (10 hair roots)	Sister: n (Bl, BM, H)<br>Maternal grandmother: n (Bl, BM, H)<br>Mother and maternal aunt not tested, neurological disorder
80.	Uziel et al. 1997 [68]	*MTATP6*	m.8993T>G	85% (Bl)	Mother: 55% (Bl)<br>Sister: 65% (Bl)<br>Maternal grandmother: not tested (deceased)<br>Three maternal aunts and one of their daughters: n (Bl)<br>Maternal great-grandmother: n (Bl)<br>Maternal grandmother's three half-

					siblings (same mother), and five of their children: n (Bl)
**One maternal tissue tested, sibling(s) tested ^**					
81.	Kirby et al. 2004 [43]	*MTND1*	m.3697G>A	80% (M), 79% (F)	Mother: 3% (Bl)<br>Two sisters: n (Bl)<br>Maternal grandmother: n (Bl)<br>Two maternal aunts: n (Bl)<br>Maternal uncle: n (Bl)
82.	Kirby et al. 2004 [43]	*MTND1*	m.3949T>C	93% (M), 88% (F), 45% (Bl)	Mother: n (Bl)<br>Two brothers: n (Bl)
83.	Bataillard et al. 2001 [100]	*MTTQ*	m.4332G>A	81% (M), n (Bl, F)	Mother: n (Bl)<br>Brother: n (Bl)
84.	Dey et al. 2000 [101]	*MTTQ*	m.4370insA	87% (M), 61% (ocular M), 23% (F)	Mother: n (Bl)<br>Two brothers: n (Bl)
85.	Vissing et al. 1998 [102]	*MTTM*	m.4409T>C	77% (M), 8% (Bl), n (H)	Mother: n (Bl)<br>Three siblings: n (Bl)
86.	Pulkes et al. 2000 [103]	*MTTY*	m.5874A>G	89% (M), n (Bl)	Mother: n (Bl)<br>Two siblings: n (Bl)
87.	Karadimas et al. 2000 [104]	*MTCO1*	m.5920G>A	61% (M), n (Bl, F)	Mother: n (Bl)<br>Sister: n (Bl)
88.	Comi et al. 1998 [105]	*MTCO1*	m.6020del5	47-68.7% (M)	Mother: n (M)<br>Three sisters: n (M)
89.	Bruno et al. 1999 [106]	*MTCO1*	m.6930G>A	27% (Bl), 75% (M), 33% (Mb)	Mother: n (Bl)<br>Sister: n (Bl)<br>Four maternal aunts: n (Bl)
90.	Bidooki et al. 2004 [107]	*MTTS1*	m.7480A>G	>80% (M), 30% (Bl)	Mother: n (Bl)<br>Sister: n (Bl, M)
91.	Campos et al. 2001 [108]	*MTCO2*	m.7896G>A	76% (M), 67% (Bl), 60% (F)	Mother: n (Bl)<br>Sister: n (Bl)
92.	Verma et al. 1997 [109]	*MTTK*	m.8313G>A	74% (M), 54% (F)	Mother: n (Bl, Pl)<br>Two brothers: n (Bl, Pl)<br>Maternal grandmother: n (Bl, Pl)<br>Maternal aunt: n (Bl, Pl)
93.	Mkaouar-Rebai et al. 2010 [44]	*MTATP8*	m.8411A>G	97% (Bl)	Mother: n (Bl)

					Sister: n (Bl)<br>NB: deceased sister with same phenotype, not tested.
94.	López-Gallardo et al. 2009 [110]	*MTATP6*	m.8618-8619insT	85% (M), 26% (Bl)	Mother : n (Bl)<br>Brother : n (Bl)<br>Maternal aunt: n (Bl)
95.	White et al. 1999 [42]	*MTATP6*	m.8993T>C	85% (Bl), 92% (F)	Mother: 77% (Bl)<br>Sister: 33% (Bl)<br>Maternal grandmother: n (Bl)<br>Two maternal aunts: n (Bl)
96.	Santorelli et al. 1993 [66]	*MTATP6*	m.8993T>G	90% (M)	Mother: 62% (Bl)<br>Brother: 79% (Bl)<br>Brother: 77% (Bl)<br>Maternal grandmother: n (Bl)<br>Two maternal aunts: n (Bl)
97.	White et al. 1999 [42]	*MTATP6*	m.8993T>G	79% (Bl), 92% (F)	Mother: 11% (Bl)<br>Sister: 59% (Bl)<br>Sister: 7% (Bl)<br>Sister: 5% (Bl)<br>Maternal grandmother: n (Bl)<br>Maternal aunt: 1% (Bl)<br>Daughter of maternal aunt 2% (Bl)
98.	White et al. 1999 [42]	*MTATP6*	m.8993T>G	80% (Bl), 74% (F)	Mother: n (Bl)<br>Sister: n (Bl)
99.	Moslemi et al. 2005 [111]	*MTATP6*	m.9191T>C	94% (M), 90% (F)	Mother: n (Bl)<br>Sister: n (Bl)
100.	McFarland et al. 2004 [71]	*MTND3*	m.10158T>C	91% (M), 81% (F), 87% (EBV-transformed lymphoblasts)	Mother: n (Bl)<br>Brother: n (Bl)<br>Maternal grandmother: n (Bl)<br>Two maternal aunts: n (Bl)
101.	McFarland et al. 2004 [71]	*MTND3*	m.10158T>C	97% (M), 95% (F)	Mother: n (Bl)<br>Sister: n (Bl)
102.	Ruiter et al. 2007 [112]	*MTND5*	m.13513G>A	64% (M), 25% (F), 26% (Bl)	Mother: n (Bl)<br>Brother: n (Bl)
103.	Tarnopolsky et al. 2013 [113]	*MTND6*	m.14487T>C	>99% (M)	Mother: n (Bl)<br>Brother: n (M)

104.	Anitori et al. 2005 [46]	*MTTE*	m.14710G>A	77% (M), trace to 14% (3 hair roots), n (Bl, F, BM, 46 hair roots)	Mother: n (Bl)<br>Three sisters: n (Bl)
105.	Pereira et al. 2007 [114]	*MTTE*	m.14724G>A	94% (M), 62% (Bl)	Mother: n (Bl)<br>Sister: n (Bl)<br>Two maternal aunts: n (Bl)
106.	Schuelke et al. 2002 [115]	*MTCYB*	m.14849T>C	69% (M), 12% (F), 6% (Bl)	Mother: n (Bl)<br>Sister: n (Bl)
107.	Andreu et al. 1999 [116]	*MTCYB*	m.15059G>A	63% (M), n (Bl)	Mother: n (Bl)<br>Sister: n (Bl)
108.	Keightley et al. 2000 [36]	*MTCYB*	m.15242G>A	87% (M), 0.7% (Bl), 92% (rectus abdominis muscle), <0.4-66%, mean 4.6% (8 hair roots), n (22 hair roots), 29-75% (F) 62% (uterus), 4-5% (ovary)	Mother: n (Bl)<br>Sister: very faint mutant bands, possibly trace amounts present (<0.2%) (Bl)
109.	Dumoulin et al. 1996 [117]	*MTCYB*	m.15615G>A	80% (M), n (Bl)	Mother: n (Bl)<br>Two sisters: n (Bl)
110.	Blakely et al. 2005 [118]	*MTCYB*	m.15699G>C	88% (M), 16% (U), 13% (Bl), 14% (H)	Mother: n (M)<br>Sister: n (M)<br>Son: n (U)
111.	Andreu et al. 1998 [119]	*MTCYB*	m.15762G>A	85% (M), n (Bl)	Mother: n (Bl)<br>Sister: n (Bl)<br>Two brothers: n (Bl)
112.	Nishino et al. 1996 [120]	*MTTT*	m.15915G>A	74% (M), 32% (F), 18% (Bl)	Mother: n (Bl)<br>Sister: n (Bl)
113.	Moraes et al. 1993 [57] and Ionasescu et al. 1994 [121]	*MTTP*	m.15990G>A	85% (M), <1% (Bl, consistently present in several experiments), n (F)	Mother: n (Bl)<br>Sister: n (Bl, F)
**Multiple maternal tissues tested, sibling(s) tested** [A]					
114.	Mancuso et al. 2004 [122]	*MTTF*	m.661G>A	91% (M), n (Bl, F, U, BM)	Mother: n (Bl, BM, U)<br>Brother: n (Bl, BM, U)<br>Daughter: n (Bl, BM, U)
115.	Coulbault et al. 2007 [123]	*MTRNR2*	m.3090G>A	~100% (M), 50% (U), n (F, H,Bl, BM)	Mother: n (U, Bl)<br>Brother: n (Bl)

116.	Campos et al. 1996 [124]	*MTTL1*	m.3243A>G	70% (M), 30% (Bl)	Mother: n (Bl, H)<br>Two sisters: n (Bl)<br>Third sister: n (M, Bl)
117.	Yamamoto 1996 [125]	*MTTL1*	m.3243A>G	89% (M), 36% (Bl)	Mother: 79% (M), 10% (Bl)<br>Maternal grandmother: n (M, Bl)<br>Maternal uncle: n (M, Bl)
118.	Ko et al. 2001 [126]	*MTTL1*	m.3243A>G	54% (M), 56% (Bl), 70% (H), 64% (BM)	Mother: 11% (Bl). 27% (H), 32% (BM)<br>Brother: 65% (Bl), 79% (H), 70% (BM)<br>Maternal grandmother: n (Bl, H, BM)<br>Maternal aunt: n (Bl, H, BM)<br>Two maternal uncles: n (Bl, H, BM)
119.	Maassen et al. 2002 [127]	*MTTL1*	m.3243A>G	18% (Bl), 55% (BM)	Mother: n (Bl, BM)<br>Three brothers: n (Bl, BM)
120.	Pallotti et al. 2014 [128]	*MTTL1*	m.3243A>G	39% (M), 32% (U), 5% (Bl)	Mother: n (Bl, U)<br>Two sisters: n (M, U)<br>Brother: n (M, U)<br>Sister: n (U)<br>Sister: n (M)<br>This sister's son and daughter: n (M)<br>Daughter: 44% (M), 29-46% (U), 10-13% (Bl), 10-67% (4 oocytes)<br>Daughter's son: 75% (U), 27% (Bl)
121.	Blakely et al. 2005 [129]	*MTND1*	m.3376G>A	98% (M), 67% (U), 18% (Bl)	Mother: n (Bl, U)<br>Two sisters: n (Bl, U)<br>Brother: n (Bl, U)
122.	Kirby et al. 2004 [43]	*MTND1*	m.3946G>A	60% (M), 45% (F), 37% (Bl)	Mother: n (Bl, U)<br>Sister: 3% (U)
123.	Taylor et al. 2002 [130]	*MTTI*	m.4267A>G	88% (M), 24% (BM), 2% (Bl), <1% (H)	Mother: n (Bl, BM, H, M)<br>Sister: n (Bl, BM, H)<br>Brother: n (Bl, BM, H)
124.	Houshmand et al. 1999 [131]	*MTTK*	m.8328G>A	10% (Bl), 57% (M), 13% (F)	Mother: n (Bl, M)<br>Three brothers and four sisters: n (Bl)<br>Daughter: n (Bl, M)
125.	Brinckmann et al. 2007 [132]	*MTTK*	m.8347A>G	31% (M), 38% (F), 37% (Bl)	Mother: n (Bl, H, BM)<br>Sister: n (Bl)
126.	Tulinius et al. 1995 [133]	*MTATP6*	m.8993T>G	94% (M), 92% (Bl)	Mother: 37% (M), 38% (Bl)

					Brother: 44% (Bl)<br>Maternal grandmother: n (M, Bl)<br>Two maternal aunts: n (Bl)<br>Maternal uncle: n (Bl)<br>Maternal great-grandmother: n (Bl)<br>Six siblings of maternal grandmother, four of their children, and one of their grandchildren: n (Bl)
127.	De Coo et al. 1996 [134]	*MTATP6*	m.8993T>G	79% (M), 74% (Bl)	Mother: n (M, Bl, F)<br>Sister: n (Bl)<br>Six maternal aunts/uncles: n (Bl)<br>Maternal grandmother: n (Bl)
128.	Degoul et al. 1997 [135]	*MTATP6*	m.8993T>G	>95% (M)	Mother: n (Bl, F)<br>Sister: n (Bl)
129.	Santorelli et al. 1997 [136]	*MTATP6*	m.8993T>G	75% (M), 72% (Bl)	Mother: n (M, Bl)<br>Brother: n (Bl)
130.	Takahashi et al. 1998 [137]	*MTATP6*	m.8993T>G	> 95% (M, U, Bl, F)	Mother: n (Bl, U)<br>Sister: n (Bl, U)<br>Maternal grandmother: n (Bl, U)
131.	Horvath et al. 2002 [138]	*MTCO3*	m.9379G>A	93% (M), n (H, Bl)	Mother: n (Bl, H)<br>Sister: n (Bl)
132.	Tiranti et al. 2000 [139]	*MTCO3*	m.9537insC	100% (M, U, Bl, F)	Mother: n (Bl, H, BM)<br>Brother: n (Bl)<br>Maternal grandmother: n (Bl)
133.	Pancrudo et al. 2007 [140]	*MTTR*	m.10406G>A	96% (M), 94% (U), 36% (BM), 29% (Bl), 0-7% (H)	Mother: n (U, BM, Bl, H)<br>Brother: n (U, BM, Bl, H)<br>Sister: n (U, BM, Bl, H)
134.	Taylor et al. 2004 [141]	*MTTH*	m.12147G>A	86% (M), 33% (U), 1% (Bl), n (H)	Mother: n (Bl, M, U, H)<br>Two sisters: n (Bl)<br>Two maternal aunts: n (Bl)<br>Two maternal uncles: n (Bl)
135.	Melone et al. 2004 [142]	*MTTH*	m.12147G>A	81% (M), 25% (Bl)	Mother: not tested (deceased, affected)<br>Sister: n (Bl, H, U)<br>Maternal grandmother: n (Bl, H, U)<br>Maternal aunt and her daughter: n (Bl, H, U)

					Maternal uncle: n (Bl, H, U)<br>Maternal grandmothers' two brothers: n (Bl, H, U)
136.	Biousse et al. 1997 [47]	*MTND6*	m.14484T>C	88% (Bl), 24% (Pl)	Mother: n (M, U, H, Bl; Pl)<br>Monozygotic twin brother: 90% (Bl), 83% (Pl)
137.	Blakely et al. 2009 [143]	*MTTP*	m.15967G>A	69% (M), 10% (U), n (Bl)	Mother: n (Bl, U)<br>Two sisters: n (Bl, U)

[a] Mother and/or sibling(s) of the individual with an apparently de novo mutation.

NB: PND (and subsequent postpartum testing of the child) is not considered testing of sibling here

Grey-colored cases: not de novo in index patient, but in other (maternal) relative

M: muscle Bl: blood U: urine F: fibroblasts H: hair BM: buccal mucosa Pl: platelets Mb: myoblasts CVS: chorionic villus sampling PGD: preimplantation genetic diagnosis
n: normal (mutation not detected)
